# Antibody engineering for SFTS treatment: frontier progress and translational challenges

**DOI:** 10.3389/fmicb.2026.1888718

**Published:** 2026-08-05

**Authors:** Xinhan Yu, Youwang Li, Shuchen Chang, Sha Li, Huaying Huang

**Affiliations:** 1 School of Integrated Chinese and Western Medicine, Nanjing University of Chinese Medicine, Nanjing, China; 2 Department of Anesthesiology, Affiliated Hospital of Nanjing University of Chinese Medicine, Nanjing, China

**Keywords:** antibody engineering, bispecific antibodies, monoclonal antibodies, nanobodies, SFTSV

## Abstract

Severe Fever with Thrombocytopenia Syndrome (SFTS), a highly fatal tick-borne infectious disease caused by SFTS virus (SFTSV), currently lacks globally approved specific antiviral drugs or vaccines, although favipiravir was approved in Japan for SFTS treatment in June 2024 based on data from non-randomized, uncontrolled single-arm trials. Antibody engineering has emerged as a core strategy against SFTSV, leveraging its advantages of high specificity, potent neutralizing activity, and engineerability. This review systematically examines the mechanisms of action and preclinical progress of monoclonal antibodies, nanobodies, and bispecific antibodies (bsAbs) for SFTS treatment. It analyzes translational challenges—including immunogenicity, manufacturing processes, cost control, drug resistance, and clinical trial design—and proposes a strategic prioritization matrix and future research directions such as multispecific antibody development, artificial intelligence (AI)-assisted design, combination therapy strategies, and international multicenter collaboration. This review aims to provide a systematic reference for the clinical translation of SFTS antibody therapeutics.

## Introduction

Severe Fever with Thrombocytopenia Syndrome (SFTS) is an acute arthropod-borne infectious disease caused by the SFTS virus (SFTSV), primarily transmitted through tick bites, with additional human-to-human transmission occurring via exposure to patients' blood or secretions. The disease manifests with acute onset and rapid progression, exhibiting a fatality rate ranging from 6.3% to 30%, which can be even higher among elderly populations and individuals with underlying medical conditions (Xilin et al., 2020). To date, no specific antiviral drugs or preventive vaccines have been approved globally, although favipiravir was approved in Japan for the treatment of SFTS in June 2024 (Fujifilm Toyama Chemical Co., 2024). This approval was based on data from non-randomized, uncontrolled single-arm trials showing improved mortality rates compared to historical controls (Suemori et al., 2021); however, the evidence level remains limited, and a confirmatory Phase III trial is currently ongoing. Clinical care primarily relies on symptomatic and supportive treatments (Chendan et al., 2026). This urgent and unmet clinical need underscores the critical importance of developing effective therapeutic interventions.

Antibody drugs, particularly monoclonal antibodies (mAbs), have become a core strategy for addressing emerging viral infectious diseases due to their high specificity, potent neutralizing activity, and engineerable effector functions (Praveen et al., 2025). Currently, multiple neutralizing antibodies against SFTSV have demonstrated promising therapeutic and prophylactic potential in preclinical studies. However, several major scientific questions and translational bottlenecks remain unresolved. These include: (i) the structural and functional differences between the two viral glycoproteins Gn and Gc, and their respective roles in viral entry and immune evasion; (ii) the distribution of neutralizing epitopes on Gn and Gc, and their conservation across different SFTSV genotypes; (iii) the structural basis of broadly neutralizing antibodies and the mechanisms by which they recognize conserved epitopes; and (iv) the translational challenges that have prevented any SFTSV antibody from advancing into clinical trials, despite encouraging preclinical data.

Although recent reviews have summarized antiviral drugs and candidate vaccines for SFTS (Jia-Chen et al., 2022; Praveen et al., 2025), a comprehensive synthesis specifically focusing on antibody engineering—ranging from structural biology insights to clinical translation strategies—has been lacking. This review systematically describes the mechanisms of action and preclinical research progress of monoclonal antibodies, nanobodies, and bsAbs for the treatment of SFTS. It integrates structural biology findings that guide epitope mapping and the design of broadly neutralizing antibodies, analyzes key translational challenges, and proposes a strategic framework to accelerate clinical development. By bridging structural insights with a translational roadmap, this review aims to provide a systematic reference for the clinical translation of antibody-based therapeutics against SFTS.

## Core experiences and lessons in antibody therapeutics for emerging viruses

Before developing antibody therapies against SFTS, it is useful to examine recent experiences with antibody drugs for other emerging viruses. This section distills key lessons from Ebola virus disease and COVID-19 that may inform SFTS antibody development.

### Ebola virus antibody therapy and the PALM trial

The PALM trial, conducted during the 2018–2019 Ebola outbreak in the Democratic Republic of Congo, represents a landmark for clinical research in outbreak settings (Sabue et al., 2019). Using a randomized controlled design, the trial compared four treatments: ZMapp (control), Remdesivir, MAb114, and REGN-EB3. Three key lessons emerged for SFTS: (i) international collaboration and pre-established planning enabled scientifically and ethically sound research during a public health emergency; (ii) adaptive design allowed early termination of inferior arms based on interim analysis, reflecting a patient-centric, rapidly iterative development paradigm; and (iii) monoclonal antibodies (particularly MAb114 and the REGN-EB3 cocktail) demonstrated significant mortality reduction, providing direct evidence for antibody therapy against highly lethal viruses (Sabue et al., 2019). For SFTS, these findings underscore the importance of evaluating candidate antibodies in the context of an outbreak, which relies on a pre-established trial framework, multi-party collaboration, and flexible study design.

### COVID-19 antibody therapy

During the COVID-19 pandemic, multiple neutralizing antibodies received emergency use authorization, but the rapid emergence of viral variants presented formidable challenges (Lanying et al., 2021). Key mutations (such as K417N/T, E484K, N501Y, etc.) in the spike protein receptor-binding domain (RBD) caused originally potent neutralizing antibodies to lose activity (Daniele and Fabrizio, 2021). This experience offers two critical insights for SFTS: (i) antibody development must incorporate broad-spectrum activity and resistance to viral escape as core design criteria; and (ii) early intervention is essential—administering antibody therapy at the onset of symptoms or using it as post-exposure prophylaxis is far more effective than treating severe cases (Evi et al., 2023). Given the rapid progression of SFTS and the narrow window for therapeutic intervention, the latter finding is particularly crucial for this disease.

### General implications for SFTS antibody development

First, proactive development strategies are needed to address emerging threats. As a tick-borne virus, SFTSV circulates among animal hosts with distinct regional and seasonal patterns (Haoliang et al., 2024). Drawing on Ebola experience, fundamental research should be strengthened during non-epidemic seasons, including screening natural neutralizing antibodies from recovered patients and identifying conserved epitopes on Gn/Gc glycoproteins.

Second, next-generation antibody technologies should pursue broad-spectrum activity and high potency. To avoid the rapid loss of efficacy caused by viral mutations—as seen with COVID-19 antibodies—SFTS antibody design must prioritize broad-spectrum coverage. This requires structural biology techniques such as cryo-electron microscopy to resolve antibody-antigen complexes and guide rational design (Kevin, 2022). Bispecific antibodies or engineered Fc regions that enhance effector functions can not only neutralize the virus but also clear infected cells (Jinge et al., 2025; Qerqez et al., 2025). Additionally, broad-spectrum antivirals targeting highly conserved viral components should be considered as combination therapies to enhance efficacy and reduce drug resistance (Marwah et al., 2023).

Third, a patient-centric precision clinical development strategy is essential. This includes defining the optimal window for antibody therapy based on SFTS natural history (viremia kinetics, cytokine storm timing); incorporating adaptive features into clinical trial designs informed by the PALM trial experience; and establishing patient stratification models using biomarkers such as baseline viral load and specific cytokine levels to achieve personalized treatment.

## Antibody engineering breakthroughs against SFTSV

While each platform has demonstrated potent neutralizing activity in preclinical models, their relative advantages and limitations—particularly with respect to clinical translation—differ substantially and are summarized below.

### Monoclonal antibodies (mAbs)

#### Early screening and characterization

Early studies constructed phage-display antibody libraries from convalescent patients and successfully isolated monoclonal antibodies with neutralizing activity. For example, Mab4-5 exhibited potent neutralizing activity and inhibited the binding of the viral glycoprotein Gn to cellular receptors (Xiling et al., 2013). Antibody Ab10 binds to Domain II and the Stem region of the Gn protein. It functions by likely inhibiting key conformational changes required for viral membrane fusion and is projected to cover over 90% of reported SFTSV isolates, demonstrating promising broad-spectrum potential (Ki Hyun et al., 2019). Antibodies targeting the Gc protein, such as C3A11, C6C1, and C4H12, also neutralize SFTSV, and their neutralizing activity is further enhanced when used in combination with anti-Gn antibodies (Kaori et al., 2024). These early studies demonstrated the neutralizing potential of Gn/Gc-targeting antibodies but suffered from low screening efficiency and limited epitope information. With advances in single-cell sequencing and structural biology techniques, structure-based antibody discovery strategies have become mainstream.

#### Structure-based high-efficiency broadly neutralizing antibodies

Current research combines single-cell sequencing, high-throughput screening, and structural biology to directly isolate potent human antibodies from convalescent B cells and guide their optimization through structural analysis. Several teams have developed representative broad-spectrum neutralizing antibodies, summarized in Table 1.

**Table 1:** Summary of characteristics of representative anti-SFTSV monoclonal antibodies.

Antibody name	Target	Epitope region	Neutralization breadth	Protection in animal models	References
S2A5	Gn	Domain I (conserved)	A-F, six SFTSV strains	Complete protection pre-/post-exposure	Xuanxiu et al. (2024)
40C10	Gn	Spatial epitope	SFTSV (genotypes C1–C4)/HRTV/GTV	Effective with delayed administration	Xiaoli et al. (2024)
1B2+4-5 or 1B2+IF6 pairing combinations	Gn	Distinct epitopes	Branches I-IV SFTSV strains	Post-exposure administration provided complete protection	Bang et al. (2025)
SD4/SD22	Gn	Hexameric well rim	SFTSV-mScarlet	Single administration achieved 100% protection	Chuansong et al. (2025)

These structure-based studies not only yielded potent candidate molecules but also laid a theoretical foundation for designing vaccines targeting conserved key epitopes (Chuansong et al., 2025). Studies indicate that monoclonal antibodies targeting Gn or Gc proteins can block infection, and antibody combinations can further enhance protective efficacy (Bang et al., 2025).

Despite these advances, most neutralizing mAbs identified to date target the Gn protein, with relatively fewer reports on Gc-specific antibodies. Whether this reflects intrinsic antigenicity differences or screening biases remains unclear, but it suggests that Gc may represent an underexplored target for therapeutic development.

#### Nanobodies

Nanobodies are single-domain antibodies derived from camelids, composed solely of a heavy-chain variable domain (VHH). They possess unique advantages including small molecular weight, high stability, ease of expression and engineering, and the ability to bind cryptic epitopes inaccessible to conventional antibodies (Xilin et al., 2020; Nehad S. et al., 2024).

Researchers immunized camels with the SFTSV Gn protein to construct a nanobody library, from which they isolated the neutralizing antibody SNB02. SNB02 effectively inhibited SFTSV replication *in vitro* and significantly mitigated virus-induced pathological changes in humanized mouse models, specifically preventing the hallmark symptom of SFTS—thrombocytopenia (Xilin et al., 2020). Studies highlight the remarkable plasticity of nanobodies. Computational modeling and AI-assisted design tools (e.g., AlphaFold2, ProteinMPNN) enable the rational optimization of nanobody affinity, stability, and humanization level (Nehad S. et al., 2024; Haoran and Yu, 2025). Their single-domain nature also facilitates their engineering into bispecific or multispecific molecules through simple genetic splicing (Mingkai et al., 2024).

Breakthroughs in nanobody screening based on heterologous immunization strategies provide highly efficient therapeutic candidates for SFTSV treatment. Wu et al. immunized alpacas using a regimen involving multiple immunizations with subtype B Gn protein followed by a booster with inactivated subtype E virus, inducing highly potent and broad-spectrum neutralizing serum. This serum exhibited significantly enhanced neutralizing activity (ID_50_) against five SFTSV subtypes (A-E) compared to single immunization approaches. A phage display library with high diversity and large capacity was constructed from the peripheral blood of immunized alpacas. Five unique nanobodies (including Nb71) with distinct complementarity-determining region (CDR) sequences were identified, exhibiting picomolar affinity binding to the Gn protein. Among them, Nb71, Nb261, Nb317, and Nb318 demonstrated potent neutralizing activity against all five SFTSV subtypes, with neutralizing capabilities significantly superior to traditional mAbs (Xilin et al., 2025). *In vivo*, Nb71 exhibited favorable pharmacokinetic properties. Following intraperitoneal injection, high serum concentrations were rapidly achieved and sustained for an extended period. In contrast, subcutaneous administration resulted in a slightly lower peak concentration but significantly prolonged the half-life.

The protective efficacy of Nb71 was further validated in two independent mouse models. In the NDG-HuPBL humanized mouse model, combined intraperitoneal and subcutaneous administration of Nb71 effectively suppressed the replication of SFTSV. In some experimental animals, viral loads were reduced to below the detection limit, and thrombocytopenia—a hallmark feature of SFTS pathophysiology—was reversed. In a lethal AG6 mouse model, early administration of Nb71 post-infection controlled viral loads, maintained normal platelet and white blood cell levels, and enabled partial survival of mice until the experimental endpoint. A serum neutralizing titer of ~4,300 ID_50_ (or ~139 μg/ml Nb71) was sufficient to confer complete protection against lethal SFTSV challenge.

Despite these encouraging results, Nb71, like most nanobody platforms, exhibits a relatively short half-life compared to full-length IgG antibodies. This inherent limitation may necessitate the application of additional engineering strategies—such as Fc fusion or albumin conjugation technologies—to further prolong its “*in vivo*” circulation time, thereby supporting its clinical translation in the treatment of SFTS.

The success of heterologous immunization in eliciting broad-spectrum nanobodies against multiple SFTSV subtypes suggests that this strategy could be generalized to other highly variable viruses. However, the short half-life of nanobodies remains a key limitation for *in vivo* application, and further engineering—such as Fc fusion or albumin conjugation—will be required to translate these candidates into clinical practice.

### Bispecific/multispecific antibodies (bsAb/msAb)

Bispecific antibodies simultaneously bind two distinct targets or different epitopes on the same target, enabling synergistic neutralization and prevention of viral escape (William, 2017; Andreas et al., 2024). SFTSV expresses two critical type I transmembrane glycoproteins on its envelope: Gn and Gc. Processed from a precursor protein, Gn primarily mediates viral attachment by binding host cell receptors, while Gc functions as a class II fusion protein that drives viral fusion with host cell membranes—the core mechanism of viral invasion. Thus, Gn and Gc constitute ideal targets for neutralizing antibodies.

Research has taken a critical step forward in addressing the therapeutic bottlenecks of SFTSV. One study screened potent human monoclonal antibodies (e.g., SF5 and SF83) targeting the Gn and Gc proteins, respectively, from convalescent patients, and used these as the foundation to construct bsAbs targeting both proteins simultaneously. Compared to the parental mAbs, these bsAbs demonstrated significantly enhanced neutralizing activity and protective efficacy both *in vitro* and *in vivo* (mouse models), achieving synergistic effects (Zhen et al., 2024). The underlying mechanism may involve simultaneous blockade of multiple critical steps, such as viral attachment and membrane fusion, or more effective inhibition of viral function through steric hindrance.

Bispecific antibodies exhibit diverse molecular formats, including IgG-based symmetric or asymmetric structures (e.g., Knob-into-Hole technology) and compact antibody fragment-based configurations [such as tandem scFv (single-chain variable fragment), DART (Dual-Affinity Re-Targeting), and sdAb (single-domain antibody) fusions; William, 2017; Xiaohan et al., 2023; Andreas et al., 2024]. Selecting the optimal bispecific antibody format for SFTS treatment requires balancing half-life, stability, production efficiency, and intended mechanism of action. Table 2 compares the core characteristics of different antibody formats and identifies their preferred clinical scenarios.

**Table 2:** Comparative analysis of core characteristics and clinical scenarios across antibody engineering formats.

Dimension	Conventional monoclonal antibody (mAb)	Nanobody	Bispecific antibody (bsAb)
Structure	Full-length IgG comprising two heavy chains and two light chains	Single-domain structure (VHH) lacking light chains, low molecular weight (~15 kDa)	Engineered structure with diverse configurations, based on IgG or antibody fragments
Target/Epitope	Typically targets a single antigenic epitope (Gn or Gc)	Capable of binding cryptic/concave epitopes	Simultaneously binds two distinct targets or epitopes
Neutralization breadth and escape resistance	Depends on epitope conservation	Targeting conserved cryptic epitopes or cocktail therapy (Xilin et al., 2025)	Theoretically advantageous, raising the viral escape barrier (Zhen et al., 2024)
Animal model efficacy	Effective in immunodeficient mice and humanized mouse models (Xuanxiu et al., 2024; Chuansong et al., 2025)	Reduced pathology and prevented thrombocytopenia in humanized mouse models (Xilin et al., 2020, 2025)	Demonstrated superior synergistic protection to parental mAbs in mouse models (Zhen et al., 2024)
Primary advantages	Technological readiness; prolonged half-life; complete effector functions	High stability; strong tissue penetration; ease of production (Nehad S. et al., 2024)	Synergistic effects; potentially high efficacy; resistance to viral escape (Zhen et al., 2024)
Potential challenges	High production costs; potentially limited tissue penetration; possible immunogenicity	Short half-life (requires fusion modification); less clinical experience than conventional antibodies (Xilin et al., 2020)	Complex molecular structures; challenging manufacturing processes; immunogenicity requires evaluation
Development readiness	Late preclinical stage	In preclinical studies	Proof-of-concept stage
Preferred clinical scenarios	Post-exposure prophylaxis and early treatment	Central nervous system infections and nebulized administration	Failure of mAb monotherapy or a high risk of viral escape

As shown in the table, this series of technologies collectively constitutes a strategic technology reserve system with tiered response capabilities. When confronting emergent SFTS outbreaks, monoclonal antibody therapy—demonstrating the highest technological readiness—can be rapidly deployed as a priority response measure. To establish durable and broad-spectrum immune protection, nanobodies and bsAbs constitute critical long-term technology reserves requiring focused development.

While bsAbs offer theoretical advantages in preventing viral escape, experimental validation in SFTS remains limited to a single proof-of-concept study (Andreas et al., 2024). Whether bsAbs outperform optimized mAb cocktails *in vivo* and whether their complex production costs can be justified for resource-limited endemic areas remain open questions that warrant further investigation.

**Figure 1:**
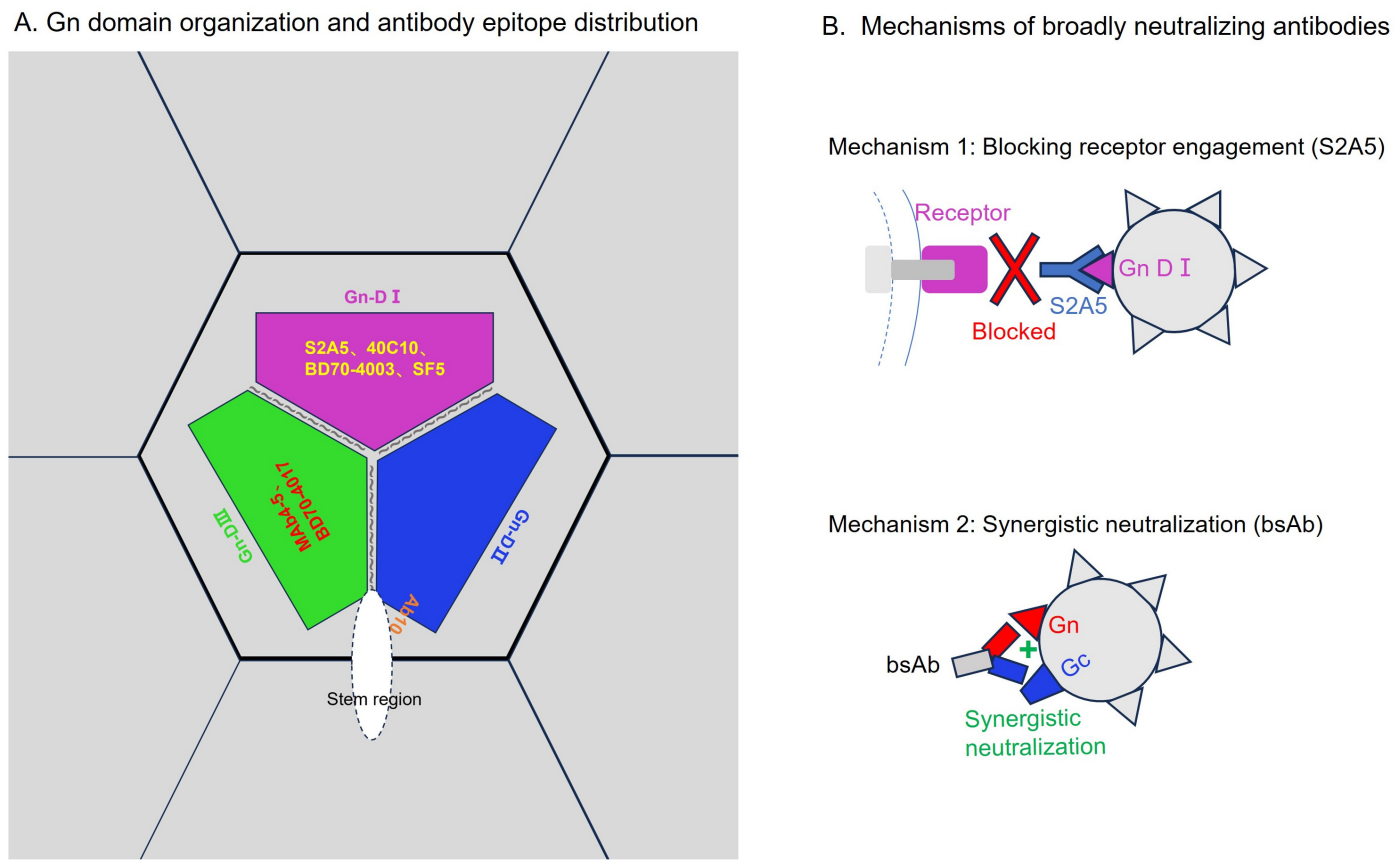
Gn domain organization and mechanisms of broadly neutralizing antibodies against SFTSV. **(A)** Schematic representation of the Gn glycoprotein ectodomain, showing the three subdomains (DI, DII, DIII) and the stem region. Representative neutralizing antibodies are positioned according to their reported epitope locations: S2A5, 40C10, BD70-4003, and SF5 target subdomain I (DI); Ab10 binds at the interface of DII and the stem region; Mab4-5 targets DIII. **(B)** Two major mechanisms of broadly neutralizing antibodies: (1) blocking receptor engagement, as exemplified by S2A5 binding to Gn DI to prevent viral attachment; and (2) synergistic neutralization through multi-epitope targeting, as exemplified by bispecific antibodies (bsAbs) that simultaneously engage Gn and Gc to enhance neutralization potency and reduce viral escape.

The structural and functional diversity of the antibody platforms discussed above—ranging from conventional monoclonal antibodies to nanobodies and bispecific antibodies—converges on the Gn glycoprotein as a primary target. To visualize the spatial relationship between antibody-binding sites and the structural organization of Gn, as well as the key neutralization mechanisms, a schematic summary is provided in Figure 1. Figure 1A illustrates the distribution of representative neutralizing antibodies across the three subdomains (DI–DIII) and the stem region of Gn, highlighting the epitope clusters that have been structurally characterized. Figure 1B depicts two major mechanisms by which broadly neutralizing antibodies achieve antiviral activity—blocking receptor engagement and synergistic neutralization through multi-epitope targeting. Together, these findings provide a structural and functional framework for understanding current antibody development efforts and guiding future engineering strategies.

## Structural biology technologies in SFTSV antibody discovery and engineering

Recent advances in structural biology have significantly accelerated the discovery and optimization of antibodies targeting viral pathogens. In particular, cryo-electron microscopy (cryo-EM) has emerged as a pivotal technique for characterizing antibody–antigen complexes at near-atomic resolution, enabling the identification of conserved protective epitopes, rational affinity maturation, and the design of broadly neutralizing antibodies (Kevin, 2022; Xiaoli et al., 2024; Xuanxiu et al., 2024; Zhen et al., 2024; Chuansong et al., 2025).

For SFTSV, cryo-EM studies have provided critical insights into the structural basis of neutralization. The Gn glycoprotein, which forms a hexagonal lattice on the viral surface, contains multiple neutralizing epitopes clustered within subdomain I and the stem region (Xuanxiu et al., 2024; Chuansong et al., 2025). Antibodies targeting these conserved regions, such as S2A5, achieve broad neutralization across different SFTSV genotypes by recognizing a conserved surface within subdomain I (Xuanxiu et al., 2024). Similarly, SD4 and SD22 target epitopes near the edge of the hexameric well, a region that appears to be structurally constrained and thus less prone to escape mutations (Chuansong et al., 2025). These structural findings have direct implications for engineering next-generation antibodies: epitopes that are both conserved and functionally constrained represent ideal targets for the design of broadly neutralizing antibodies and provide a rational basis for constructing bsAbs capable of simultaneously binding two distinct protective epitopes (Zhen et al., 2024).

Complementing cryo-EM, single-cell B cell sequencing enables the direct isolation of potently neutralizing antibodies from convalescent SFTSV patients, thereby bypassing the need for hybridoma or phage display screening (Kaori et al., 2024; Chuansong et al., 2025). The integration of high-throughput antibody screening platforms with structural characterization has accelerated the identification of antibodies with favorable epitope profiles and broad neutralizing capacity. Artificial intelligence-assisted protein design tools, such as AlphaFold2 and ProteinMPNN, have further expanded the engineering toolkit by enabling in silico prediction of antibody–antigen interactions and rational optimization of antibody affinity and stability (Nehad S. et al., 2024; Haoran and Yu, 2025).

### Structural insights from representative anti-SFTSV antibodies

Cryo-EM and X-ray crystallography studies have provided high-resolution structural information for several representative anti-SFTSV antibodies, revealing the spatial distribution of protective epitopes and the molecular basis of neutralization.

#### Epitope distribution on Gn and Gc

The Gn glycoprotein comprises three major subdomains (DI–DIII). Protective epitopes have been identified across multiple subdomains, with DI emerging as a particularly important region. The broadly neutralizing antibody S2A5 recognizes a conserved epitope within Gn DI, enabling cross-neutralization across all six SFTSV genotypes (A–F; Xuanxiu et al., 2024). Cryo-EM analysis of an optimized S2A5 variant bound to SFTSV virions revealed that enhanced neutralization potency correlates with improved binding to recombinant Gn and intact virions, likely facilitated by crosslinking adjacent Gn subunits on the viral surface (Xuanxiu et al., 2024). Similarly, the human monoclonal antibody SF5, which also targets Gn DI, has been structurally characterized and exhibits potent protective efficacy in mouse models (Zhen et al., 2024). In contrast, antibodies targeting Gn DIII, such as Mab4-5, show potent *in vitro* neutralization but limited *in vivo* protection, suggesting that epitope location and binding mode may influence protective efficacy (Xiling et al., 2013). For the Gc glycoprotein, antibody SF83 binds to Gc DII, and structural analysis has informed the design of bsAbs that simultaneously engage both Gn and Gc (Zhen et al., 2024).

#### Epitope conservation and broad neutralization

Comparative analysis of protective antibodies against SFTSV and RVFV indicates that neutralizing epitopes are predominantly located on Gn DI across these phleboviruses, suggesting that this region may represent a conserved hotspot for protective antibody responses (Zhen et al., 2024). The SD4 and SD22 antibodies recognize epitopes near the hexameric well rim of Gn, a structurally constrained region that appears less permissive to escape mutations (Chuansong et al., 2025). The Ab10 antibody binds to Gn DII and the stem region, with structural analysis suggesting that it may inhibit membrane fusion by blocking key conformational changes required for viral entry (Ki Hyun et al., 2019).

#### Implications for rational design

These structural findings provide a molecular framework for engineering next-generation antibodies. The identification of conserved, structurally constrained epitopes on Gn DI and the hexameric well rim offers rational targets for broad-spectrum antibody design (Xuanxiu et al., 2024; Chuansong et al., 2025). Furthermore, the close spatial proximity of epitopes on Gn (SF5) and Gc (SF83) enabled the construction of bsAbs that simultaneously engage both glycoproteins, achieving synergistic neutralization and reduced viral escape (Zhen et al., 2024). This structure-guided approach demonstrates how epitope mapping can directly inform the design of multispecific therapeutics with enhanced potency and resistance to viral mutations.

The convergent targeting of neutralizing antibodies toward these structurally constrained sites is not coincidental. These regions are characterized by functional constraints that restrict escape mutations, suggesting that they may represent structurally vulnerable sites on the viral surface—a feature that could be leveraged for the rational design of vaccines and therapeutic agents. The structure-guided paradigm exemplified by bsAbs targeting Gn/Gc provides a template for the rational design of antibodies against other emerging phleboviruses.

## SFTSV-specific scientific questions and therapeutic implications

### Structural, functional, and immunological differences between Gn and Gc

SFTSV encodes two envelope glycoproteins, Gn and Gc, which form heterodimers on the viral surface and mediate distinct steps of viral entry (Du et al., 2023). Gn is a type I transmembrane protein responsible for receptor attachment and constitutes the primary target of neutralizing antibodies (Xuanxiu et al., 2024; Wang et al., 2026). Its ectodomain comprises three subdomains (DI, DII, and DIII), with DI characterized by high surface exposure and believed to harbor critical interfaces for host receptor engagement. DII functions as a β-connector structure, while DIII forms a structural “cap” that partially shields the fusion loop of Gc, thereby regulating the timing of membrane fusion (Wang et al., 2026).

In contrast, Gc is a class II fusion protein that orchestrates pH-dependent fusion of viral and endosomal membranes. Structurally, Gc adopts a three-domain architecture (DI, DII, DIII) in its postfusion conformation, with domain II containing the fusion loops that insert into the host membrane (Halldorsson et al., 2016). Conformational rearrangements of Gc from prefusion to postfusion states are triggered by acidic pH, involving a histidine switch mechanism (Halldorsson et al., 2016). Notably, the preservation of these domains across diverse SFTSV clades makes Gn and Gc attractive targets for therapeutic development, although Gn appears to be the immunodominant target for neutralizing antibodies (Wang et al., 2026).

### Distribution of neutralizing epitopes on Gn and Gc

Recent deep mutational scanning (DMS) and cryo-EM studies have systematically delineated the antigenic landscape of the Gn head domain. Gn-specific mAbs from convalescent SFTSV survivors have been classified into eight distinct epitope groups (IA–ID, IIA, IIB, IIIA, and IIIB), among which four groups (IA, ID, IIIA, and IIIB) confer neutralization (Wang et al., 2026). Groups IA, IC, and ID are localized to domain I; group IA antibodies (e.g., BD70-4003) target a surface-exposed region involving residues D159, G161, S163-S164, and L166. Groups IIA and IIB target domain II but often exhibit poor neutralizing activity, most likely due to the domain's less accessible orientation on the mature virion. Group IIIA (e.g., BD70-4017) and IIIB antibodies target domain III, with group IIIB including the well-characterized Mab4-5 that recognizes an epitope involving residues E271, G282, C287, K288, and C292 (Zhen et al., 2024). Importantly, the most potent neutralizing activity is concentrated in groups IA and IIIA, followed by IIIB and ID.

In contrast, Gc-specific mAbs demonstrate restricted neutralizing activity compared to Gn-specific antibodies. This is consistent with the structural observation that Gn forms a densely packed lattice on the viral surface, while Gc is partially shielded by Gn in the prefusion conformation. The structural basis for this differential immunogenicity has been elucidated by cryo-EM studies showing that Gn and Gc heterodimers assemble into higher-order pentameric and hexameric structures, creating cryptic epitopes that are only accessible during intermediate states of virus entry (Du et al., 2023).

### Antigenic variation among SFTSV genotypes and escape hotspots

SFTSV exhibits substantial genetic diversity, with multiple genotypes co-circulating across endemic regions. Phylogenetic analysis based on the M segment has identified two major lineages (L1-Chinese and L2-Japanese-Korean) comprising 13 genotypes (I–XIII). A broader classification scheme based on the complete Gn/Gc sequence further divides SFTSV isolates into genotypes A–F, which are nested within the I–XIII framework (Leng et al., 2026). This genetic diversity poses challenges for broad-spectrum antibody development.

Viral escape from neutralizing antibodies is primarily driven by mutations in the Gn head domain, particularly within the neutralizing epitope groups. Studies have identified that multi-site mutations in Gn, including I323V and K619R, can confer complete resistance to neutralizing antibody pressure. Notably, weakened invasion resulting from site mutations (e.g., D170N reducing CCR2 binding affinity) can be compensated by other mutations (I323V/K619R) that enhance immune evasion, suggesting that SFTSV employs a trade-off mechanism balancing entry efficiency and immune escape (Wang et al., 2025). This highlights the importance of targeting conserved, functionally constrained epitopes to reduce the risk of escape.

### Mechanisms of broadly neutralizing antibodies and implications for therapeutic development

Broadly neutralizing antibodies against SFTSV share several common mechanistic features. First, they typically target conserved epitopes on Gn DI or DIII that are essential for viral entry, such as the domain I epitope recognized by S2A5 and BD70-4003 (Wang et al., 2025). These antibodies effectively block the interaction between Gn and host cell receptors, preventing viral attachment. Second, some broadly neutralizing antibodies, such as the optimized S2A5 variant, achieve enhanced neutralization potency through crosslinking adjacent Gn subunits on the viral surface, thereby stabilizing the prefusion conformation and preventing membrane fusion. Third, antibodies targeting multiple epitope groups can be combined to reduce the risk of viral escape, as demonstrated by the synergistic protection observed with antibody cocktails (Wang et al., 2026).

The identification of structurally constrained, conserved epitopes on Gn DI (e.g., the surface-exposed region targeted by group IA antibodies) and the hexameric well rim region (targeted by SD4/SD22) provides a rational basis for therapeutic design (Xuanxiu et al., 2024; Wang et al., 2026). Furthermore, the close spatial proximity of epitopes on Gn and Gc has informed the construction of bsAbs that simultaneously engage both glycoproteins, achieving synergistic neutralization and reduced viral escape (Zhen et al., 2024). These structure-guided approaches demonstrate how epitope mapping can directly inform the design of next-generation antibody therapeutics with improved breadth, potency, and resistance to viral mutations.

## Deepening preclinical studies and bottlenecks

Advancing promising candidate antibodies into clinical trials necessitates robust, systematic, and translatable preclinical studies. However, the field of SFTS antibody therapy currently faces critical bottlenecks involving in-depth understanding of antibody mechanisms of action, appropriate selection and evaluation of animal models, and comprehensive awareness of risks such as immunogenicity and pharmacokinetics.

### Multidimensional analysis of mechanisms of action and corresponding design strategies

Understanding how antibodies combat SFTSV is fundamental for rational drug design, predicting clinical efficacy, and mitigating risks such as antibody-dependent enhancement. Current research has moved beyond solely evaluating neutralizing activity to a multifaceted investigation of mechanism.

First Dimension: Targeting viral proteins and key epitopes. Antibodies primarily target two structural proteins of SFTSV: the nucleocapsid protein (N protein) and the envelope glycoproteins (Gn/Gc). Although antibodies against the N protein generally lack direct virus-neutralizing capability, their high abundance in convalescent patients indicates a significant role in humoral immunity. Research has identified at least four distinct antigenic epitopes on the N protein, providing a molecular basis for developing N protein-based diagnostic tools and potential therapeutic strategies that may function through alternative mechanisms, such as modulating immune responses (Li et al., 2012). In contrast, antibodies targeting glycoproteins Gn and Gc constitute the primary source of neutralizing antibodies, directly blocking viral attachment and cellular entry (Zhen et al., 2024). Notably, epitopes within the Gn subdomain I (Subdomain I) have been identified as critical regions for eliciting protective antibodies. This finding holds significance not only for SFTSV but also provides insights for Rift Valley fever virus (RVFV), another member of the Bunyavirales family (Zhen et al., 2024). This suggests that antibodies targeting such conserved, critical epitopes could represent a breakthrough for developing broad-spectrum therapies.

Second dimension: Synergistic neutralization mechanisms and steric hindrance. Traditional monoclonal antibodies block virus-receptor interactions by binding to a single epitope. The engineered design of bsAbs introduces mechanistic innovations. By integrating the binding domains of Gn-targeting and Gc-targeting antibodies into a single molecule, bsAbs simultaneously engage two distinct glycoproteins on the viral surface. This dual binding creates steric hindrance, significantly enhancing neutralization capacity. Such synergistic effects demonstrate markedly superior efficacy compared to simple mixtures of parental antibodies (Zhen et al., 2024). This suggests that next-generation antibody design should move beyond single-epitope limitations and consider multivalent, multi-target strategies to maximize neutralizing potency and mitigate viral escape.

The third dimension: Fc-mediated effector functions. As previously discussed in Section “General Implications for SFTS Antibody Development,” engineering the Fc segment to enhance effector functions like ADCC represents a key direction for next-generation antibody design. However, for SFTSV, the specific contribution of Fc-mediated antibody—dependent cellular phagocytosis (ADCP)—another critical effector function-in clearing infected cells, along with its potential to induce harmful inflammatory responses, remains a knowledge gap. Future studies must incorporate these Fc effector functions as core evaluation parameters, which can be precisely modulated through Fc engineering (e.g., by adjusting affinity for FcγRs) to balance efficacy and safety. Fc engineering strategies, including modifications that enhance binding affinity to Fcγ receptors (FcγRs) or improve neonatal Fc receptor (FcRn)-mediated recycling, can precisely modulate ADCC/ADCP activity and extend antibody half-life (Ko et al., 2021; Ramdani et al., 2022).

### Evaluation and selection of animal model spectrums

Selecting appropriate animal models is a prerequisite for objectively evaluating the *in vivo* protective efficacy of antibodies. While a gold standard model is currently lacking in SFTS research, a spectrum of models has been established, each with specific applications and limitations (Nathen et al., 2020; Chenghao et al., 2025). Moving beyond traditional empirical approaches, employing systematic frameworks for model evaluation and selection is key to enhancing the translational value of preclinical data.

Interferon-deficient mouse models, such as A129 (IFN-α/β receptor knockout), IFNAR^−/−^, and STAT2^−/−^ mice, are highly susceptible to SFTSV and can develop lethal infections, making them suitable for evaluating the survival protection efficacy of antibodies (Nathen et al., 2020). However, these models have limitations in quantitatively evaluating Fc-mediated effector functions such as ADCC and ADCP. Although they retain effector cells that express Fc receptors and can reproduce certain Fc-dependent phenomena—including antibody-dependent enhancement (ADE)—under specific viral infection conditions, the interferon-deficient environment may not faithfully recapitulate physiological Fc effector responses. Additionally, these models do not fully simulate the complexity of the human immune response network, which constrains the translational relevance of the data (Bronwyn and Shuangyi, 2021).

Regarding immunocompetent animals, adult C57BL/6 mice exhibit mild symptoms post-infection, resembling mild human infections (Nathen et al., 2020). This model is suitable for studying the prophylactic effects or early therapeutic efficacy of antibodies in intact immune systems, but is of limited value for evaluating therapeutic effectiveness against severe infections.

The ferret model shows considerable promise, exhibiting age-dependent disease severity where older ferrets develop high-mortality symptoms including high fever and thrombocytopenia post-infection, closely aligning with human clinical manifestations (Nathen et al., 2020; Su-Jin et al., 2022). This model serves as an excellent system for evaluating antibody efficacy in ameliorating severe pathological outcomes.

Humanized mouse models, established through transplantation of human hematopoietic stem cells or immune cells, can partially recapitulate the human immune system and have been employed to evaluate the efficacy of therapies including nanobody SNB02 (Xilin et al., 2020). This model is particularly suitable for investigating the pharmacodynamics and mechanism of action of antibodies within a humanized immune environment.

In summary, model selection should be based on research objectives. Immunodeficient mice are suitable for assessing direct neutralization and survival protection. For evaluating comprehensive therapeutic efficacy (including immunomodulation) in environments more closely resembling the human body, ferret or humanized mouse models offer greater value. Despite the availability of the aforementioned animal models, none fully recapitulate all aspects of the pathophysiology observed in human SFTS. Immunodeficient and interferon-deficient models lack critical components of the adaptive immune response, thereby limiting their utility for evaluating Fc-mediated effector functions. In contrast, immunocompetent models fail to develop the full spectrum of severe disease manifestations. The absence of a gold-standard model introduces uncertainty when translating preclinical efficacy data for clinical trial design and underscores the ongoing necessity for model development and cross-model validation.

### Key limitations in preclinical evidence systems

Regarding immunogenicity risks, therapeutic antibodies may induce the production of anti-drug antibodies (ADAs) in humans, leading to accelerated drug clearance, reduced efficacy, or allergic reactions (Anshu et al., 2016; Zuben et al., 2023). The degree of humanization, aggregation propensity, and other factors of SFTS antibodies (particularly nanobodies and engineered bsAbs) require rigorous preclinical evaluation (Anshu et al., 2016; Yingda et al., 2018). Current methodologies are evolving from singular to multifaceted approaches: for instance, utilizing bioinformatics tools (such as IEDB) to scan antibody sequences and predict potential T-cell epitopes enables sequence optimization through “de-immunization” during early design stages, thereby mitigating risks (Chantal and Sivan, 2024). *In vitro* cell experiments can also be performed using human peripheral blood mononuclear cells (PBMCs) or dendritic cells (DCs) in a co-culture system with CD4+ T cells to assess the ability of antibodies to trigger T-cell activation and proliferation (Robin et al., 2024).

Additionally, a comprehensive risk assessment is required to establish an integrated evaluation plan covering product-related factors (sequence, aggregates, impurities), patient-related factors, and clinical risks.

Regarding pharmacokinetic properties, the half-life of antibodies *in vivo* directly affects the dosing regimen and therapeutic durability. Although Fc region engineering—such as enhancing binding affinity to the neonatal Fc receptor (FcRn)—is a well-established strategy for extending half-life, small antibody formats like nanobodies generally exhibit short half-lives and require alternative modification approaches. Main strategies include: fusing antibody fragments with the Fc segment of IgG or human serum albumin, thereby leveraging the FcRn recycling mechanism or the long half-life of albumin to significantly prolong circulation time (Eun Byeol et al., 2024); or conjugating polyethylene glycol chains to antibody fragments, thereby increasing their hydration radius to reduce renal filtration and extend half-life (Huanbo et al., 2019). Therefore, during preclinical development, pharmacokinetic characteristics must be systematically evaluated and optimized according to the antibody format and intended dosing regimen.

It is noteworthy that current understanding of the immunogenicity risks associated with novel antibody formats—particularly nanobodies and bsAbs—remains largely derived from experience with conventional monoclonal antibodies. Whether the predictive value of these established detection methods is applicable to engineered antibody formats featuring non-natural structures or multispecific binding properties has yet to be fully substantiated. There is an urgent need for platform-specific immunogenicity studies to validate current risk assessment approaches.

## Toward clinical translation: translational science and development strategy

Translating SFTS antibody therapy from laboratory discoveries to accessible bedside treatments constitutes a systematic process fraught with scientific and technical challenges. This section will delve into the core components of this translational pathway, encompassing goal-oriented clinical development design, novel combination paradigms to address complex pathology, and critical bottlenecks that must be overcome to achieve industrialization and broad accessibility.

### End-to-end clinical development pathway design

The clinical development of SFTS antibody therapeutics must anchor in the ultimate goal—reducing the severe disease and fatality rates in endemic areas—and design efficient, pragmatic, and ethically sound trial pathways accordingly. Lessons from antibody therapies for other emerging viral diseases, particularly the PALM trial for Ebola, are instructive.

A typical clinical development pathway for SFTS antibodies may include the following stages, with its core objective aligning with traditional antibody drug development: first assessing safety, followed by exploration and confirmation of efficacy.

Phase I clinical trials primarily evaluate the safety, tolerability, pharmacokinetics (PK), and pharmacodynamics (PD) characteristics of antibodies in humans, and determine the Maximum Tolerated Dose (MTD) or Recommended Phase II Dose (RP2D). This phase typically enrolls 20–100 healthy volunteers or SFTS patients using a dose-escalation design (e.g., the “3+3” rule) to identify a safe and effective dosing regimen while minimizing patient exposure to subtherapeutic or toxic doses. For SFTS antibodies, special attention should be paid to their potential to trigger ADE or other unintended immune responses. Regarding dose design, estimation can proceed as follows: Obtain the effective protective dose (ED_90_) and pharmacokinetic parameters (half-life, Cmax, AUC) from animal models (e.g., mice, ferrets); Convert to human equivalent dose using allometric scaling. The formula—Human dose (mg/kg) = Animal dose (mg/kg) × (Animal weight/Human weight)^0.33^–has been widely applied for small-molecule drugs. However, for monoclonal antibodies and other biologics, dose translation is more appropriately guided by pharmacokinetic/pharmacodynamic (PK/PD) modeling, exposure matching, target-mediated drug disposition, and the minimal anticipated biological effect level (MABEL) approach. The allometric scaling equation may serve as a preliminary reference for antibody therapeutics but should not be considered a standard method, as it does not account for target-mediated clearance, non-linear pharmacokinetics, or species-specific differences in FcRn binding affinity—factors critical to antibody disposition in humans (Shannon et al., 2007; Anroop and Shery, 2016; Anroop et al., 2018); Considering SFTSV viremia kinetics (typically peaking 7–10 days post-symptom onset; Zhen-Dong et al., 2016), the antibody half-life should at least cover the active viral replication period (Jun et al., 2014). A target half-life of ≥7–14 days is recommended (Liming, 2017); For formats with short half-lives such as Nanobodies, Fc fusion or albumin fusion may be employed to extend duration beyond 7 days (Ole et al., 2021), or a “loading dose plus multiple maintenance doses” regimen can be implemented (Liming, 2017).

Phase II clinical trials: this stage focuses on initial efficacy assessment and further safety confirmation, typically enrolling 100–300 SFTS patients. In adaptive trial designs, sample sizes require dynamic adjustment based on interim analysis results to maximize trial efficiency (Josephine et al., 2025). Given the rapid progression of SFTS, Phase II trials should focus on exploring early therapeutic windows. Preclinical and observational data indicate that early intervention—ideally within the first few days of symptom onset—may be critical. This time window aligns with the typical viremia kinetics of SFTSV, during which viral load peaks (around day 6 after symptom onset) and the host inflammatory response escalates (Zhen-Dong et al., 2016; Chen et al., 2026). Trial designs may employ randomized controls, with primary endpoints including viral load reduction rate, time to clinical symptom alleviation, or proportion progressing to severe disease. Another key objective at this stage is identifying predictive biomarkers of efficacy, such as specific cytokine levels or viral RNA clearance kinetics, to guide subsequent trials and clinical application (Yulan et al., 2012). Future antibody trials should incorporate biomarkers for patient stratification and early efficacy assessment. Baseline viral load, platelet count, and inflammatory markers such as Interleukin-6 (IL-6) and IL-10 may help identify patients most likely to benefit from antibody therapy. Laboratory indicators reflecting disease severity, including lactate dehydrogenase and markers of coagulation dysfunction, could serve as early surrogate endpoints for treatment response.

Phase III clinical trials: this confirmatory phase aims to verify the efficacy of antibody therapy in reducing SFTS fatality rates or preventing severe disease progression. These trials require large patient cohorts and employ a randomized, double-blind, placebo-controlled design, with stratification based on disease severity. Primary endpoints typically include all-cause mortality or composite endpoints such as death plus intensive care requirement. Given that severe SFTS patients often experience multiple organ failure and cytokine storm (Yulan et al., 2012), the trial protocol must detail synergistic approaches combining antibody therapy with standard supportive care (including respiratory support and coagulation correction) while closely monitoring immunomodulatory effects.

Furthermore, innovative trial designs such as seamless Phase I/II or II/III designs can consolidate stages to accelerate development, particularly when addressing public health emergencies. Regardless of the design adopted, the entire development pathway must be patient-centered and fully consider feasibility for implementation in SFTS-endemic regions (often rural areas with limited medical resources).

It is also important to recognize that the clinical development pathways for nanobodies and bsAbs may require trial designs distinct from those used for conventional mAbs. Their unique pharmacokinetic and pharmacodynamic characteristics—such as target-mediated drug disposition and potential immunogenicity—may require different dose selection strategies, sampling schedules, and safety monitoring plans. These considerations should be incorporated early in trial planning, rather than being extrapolated directly from experience with mAbs.

### The new paradigm of combination therapy

The pathogenic mechanism of SFTS is complex, involving a dual impact from direct viral damage and the host's excessive immune response (cytokine storm; Min et al., 2022). Single-mechanism antibody therapy may be insufficient to address all clinical scenarios, particularly for patients who have progressed to severe stages. Establishing a new paradigm for combination therapy is essential to enhance efficacy, prevent drug resistance, and reduce dosage and toxicity.

Combining antibodies with direct-acting antivirals constitutes a “block and clear” strategy. Antibodies primarily neutralize extracellular free virus, preventing new cell infections; while small-molecule antivirals (e.g., nucleoside analogs) enter cells to inhibit viral RNA replication. For instance, combining neutralizing antibodies with favipiravir may theoretically inhibit viral replication and clear viral particles simultaneously, achieving a more rapid and comprehensive virological response while reducing the emergence of drug-resistant strains due to monotherapy pressure. Notably, Favipiravir has demonstrated potential in inhibiting viral replication for SFTS treatment, including an IC_50_ of 6.0 μM *in vitro*, 100% survival protection through early administration in animal models, and accelerated viral clearance in clinical studies (Xinhan et al., 2026). In June 2024, favipiravir was approved in Japan for the treatment of SFTS. However, the evidence base remains limited, and further validation through Phase III randomized controlled trials is required to establish the optimal therapeutic regimen, particularly for use in severe cases.

Combining antibodies with immunomodulators to target cytokine storms—a key driver of severe SFTS—constitutes a critical therapeutic strategy. This approach facilitates viral clearance by antibodies while rationally employing immunomodulators to control excessive inflammatory responses. For example, combination therapy with glucocorticoids or specific anti-inflammatory cytokine inhibitors effectively controls hyperinflammatory responses concurrent with antibody-mediated viral clearance (Jia-Chen et al., 2022).

The combined use of antibodies targeting distinct epitopes—such as monoclonal antibodies or nanobodies directed against different epitopes—can broaden the neutralization spectrum and prevent viral escape. Although bsAbs can functionally mimic this effect, their concept is fundamentally different from that of conventional combination therapy. Rather than administering two separate antibodies simultaneously, bsAbs are artificially engineered multispecific molecules that simultaneously bind two different targets or epitopes within a single entity. Their design, molecular formats, and application against SFTSV have been discussed in detail in the Section entitled “Bispecific/Multispecific Antibodies.” As an engineered platform, bsAbs offer advantages over two separate antibodies, including synergistic binding and potentially reduced production complexity. Nevertheless, their development presents unique challenges in terms of production, stability, and immunogenicity evaluation.

### Industrialization and accessibility challenges

Even if antibodies prove effective in clinical trials, their ultimate ability to reach patients, especially in resource-limited rural areas where SFTS is primarily prevalent, depends on industrialization capabilities and accessibility strategies. This involves challenges across the entire chain from production to distribution.

In large-scale production, challenges include cell line stability, non-linear responses during process scale-up (such as deviations in metabolite accumulation caused by changes in oxygen mass transfer), and control of product heterogeneity (e.g., glycosylation, charge variants; Petra et al., 2014; Mikhail et al., 2025). The production processes for complex formats such as bsAbs (e.g., ensuring correct chain pairing) and purification present greater difficulties (Vicki et al., 2019). Consequently, the “developability” characteristics of candidate antibodies—including their propensity for aggregation, viscosity, and chemical and physical stability—are critical to predict and optimize early in development to avoid late-stage failures (Yingda et al., 2018).

Regarding access strategies, factors such as drug production costs, storage conditions (particularly cold chain requirements), and administration methods (intravenous infusion/subcutaneous injection) are important considerations for the final stages to ensure accessibility in SFTS-endemic areas, often resource-limited rural settings. From a health economics perspective, cost-effectiveness analysis of antibody therapy can be incorporated into early-stage research and development (R&D) decisions. Taking SFTS-endemic regions in China as an example, the per capita direct medical costs for SFTS patients are approximately $5,800–6,600 (approximately RMB 42,000–48,000), accounting for over 76% of the annual per capita disposable income (Lei et al., 2019). For critically ill patients requiring continuous renal replacement therapy or ventilator support in China, costs may escalate to over 150,000 yuan. If antibody therapy can reduce severe case conversion rates by 50% and lower fatality rates from 15% to 5%, a simple break-even analysis—based on current average direct medical costs for SFTS patients in China of approximately RMB 42,000–48,000 (Lei et al., 2019) and assuming no additional costs beyond the drug itself—suggests that a single-course production cost of approximately RMB 20,000–40,000 could be offset by the savings from reduced severe cases and fatalities. This illustrative calculation is intended to provide a preliminary reference rather than a formal cost-effectiveness threshold.

In conclusion, the translational success of SFTS antibody therapy depends not only on scientific clinical validation but crucially on establishing a resilient, efficient, and affordable industrialization and distribution system. This will likely require close collaboration among developers, regulatory agencies, public health departments, and local communities to bridge the “last mile” from laboratory to bedside—particularly to remote farming households.

## Conclusions and perspectives

### Key conclusions

Antibody engineering offers a promising avenue for the treatment of SFTS. This review draws three core conclusions. First, targeting the viral surface glycoproteins Gn and Gc has proven effective, while structure-guided rational design can further optimize the neutralizing breadth, potency, and mechanism of action of antibodies. Second, the translation from bench to bedside continues to encounter persistent challenges, including the lack of an ideal animal model, incomplete understanding of Fc-mediated effector functions, and the critical influence of treatment timing, antibody dosage, and viral escape on clinical efficacy. Third, successful translation requires a systematic strategy that extends beyond molecular optimization to encompass clinical trial design, combination therapy, and the development of affordable production and distribution systems. These findings underscore that advancing SFTS antibody therapeutics necessitates not only the discovery of novel antibodies but also a coordinated roadmap from preclinical development to clinical translation.

### SFTS antibody R&D prioritization matrix

To facilitate rational decision-making under resource-limited conditions, we evaluated the translational readiness of three major antibody platforms—monoclonal antibodies, nanobodies, and bsAbs—based on multiple development-related dimensions, including manufacturing maturity, regulatory experience, clinical validation status, and key translational barriers (Table 3).

**Table 3:** Translational readiness assessment of antibody platforms for SFTS.

Platform	Manufacturing maturity	Regulatory experience	Clinical validation status	Key translational barriers	Priority level
Monoclonal antibodies (mAbs)	High: well-established CHO cell-based platforms, standardized purification processes	Extensive: multiple approved products; clear regulatory pathways for neutralizing antibodies	Proven: clinical proof-of-concept established for viral infections	Limited: manufacturing and regulatory pathways well characterized	Highest (prioritize for near-term clinical trials)
Nanobodies	Medium: E. coli/yeast expression feasible; but scale-up and consistency less established	Limited: few approved products; regulatory standards for novel formats still evolving	None: no SFTSV candidate has entered clinical development	Half-life extension, manufacturing scalability, regulatory qualification	Medium (accelerate translational research)
Bispecific antibodies (bsAbs)	Low-to-Medium: complex production (chain pairing, purity); higher costs	Limited: few approved products; diverse formats complicate standardization	Minimal: only one proof-of-concept study for SFTS; clinical validation lacking	Production complexity, immunogenicity, cost-effectiveness	Medium (further validation required)

As summarized in Table 3, monoclonal antibodies currently demonstrate the highest level of translational readiness. This platform benefits from well-established manufacturing processes, extensive regulatory experience, and validated clinical proof-of-concept for neutralizing antibodies targeting other viral pathogens. Therefore, monoclonal antibodies should be prioritized for near-term clinical trials for SFTS.

Nanobodies and bsAbs offer notable theoretical advantages—nanobodies exhibit superior tissue penetration, stability, and manufacturability, whereas bsAbs have the potential to target multiple epitopes and possibly reduce viral escape. However, both platforms face significant unresolved translational barriers. For nanobodies, no candidate has yet entered clinical development for SFTSV, and challenges remain regarding half-life extension, manufacturing scalability, and regulatory qualification. For bsAbs, only one proof-of-concept study for SFTS has been reported to date, and their complex production processes and immunogenicity concerns may limit feasibility in resource-limited endemic regions.

Artificial intelligence-assisted design and process optimization are enabling tools rather than therapeutic platforms. These technologies are discussed separately as complementary strategies to accelerate discovery and clinical translation, but they were not incorporated into the platform prioritization framework.

This evaluation indicates that the most mature platform—monoclonal antibodies—is currently best suited for near-term clinical advancement, whereas nanobodies and bsAbs warrant continued strategic investment to address their remaining translational challenges.

### Critical translation barriers and priority actions

Accelerating the translation of SFTS antibodies from basic research to clinical application necessitates a focus on four interrelated priorities.

First, patient selection and treatment timing are critical. The success of clinical trials depends on identifying the optimal therapeutic window—typically within 72 h of symptom onset—and utilizing biomarkers, such as baseline viral load, platelet count, and inflammatory cytokines (e.g., IL-6, IL-10), to stratify patients most likely to benefit. This approach maximizes statistical power within resource-limited, small-scale trials while minimizing the risk of exposing patients to ineffective treatments.

Second, cost-effective production processes and regional accessibility are essential. The affordability of antibodies in endemic countries must be considered during their development. Relevant strategies include: (i) prioritizing antibody formats compatible with low-cost microbial or mammalian expression systems; (ii) developing thermostable formulations to reduce reliance on the cold chain; and (iii) establishing partnerships with regional manufacturers early in development to facilitate technology transfer and tiered pricing. These measures are crucial for bridging the “last mile” from regulatory approval to patient access in rural East Asia.

Third, combination treatment regimens and adaptive trial frameworks are required. Given the dual pathophysiology of SFTS—a combination of direct viral cytopathic effects and immunopathological damage—future treatment regimens should combine neutralizing antibodies with host-directed therapies [e.g., Janus kinase (JAK) inhibitors to modulate cytokine storms] or direct-acting antivirals (e.g., favipiravir). Adaptive platform trials, similar in design to the PALM study, offer a practical model for simultaneously evaluating multiple treatment arms during an outbreak, enabling the rapid identification of effective combination therapies while conserving limited patient cohorts and trial resources.

Fourth, enabling technologies to accelerate the discovery process. Artificial intelligence-assisted design tools, such as AlphaFold2 and ProteinMPNN, are playing an increasingly critical role in antibody discovery and optimization. These tools are particularly valuable for predicting antigen-antibody interactions and facilitating the design of broadly neutralizing antibody candidates. Although these computational methods are not therapeutic modalities themselves, they have the potential to significantly reduce both the time and cost associated with lead compound identification. Therefore, they should be integrated into early-stage research and development pipelines.

## References

[B1] Andreas V. M. Lasse E. P. Peter K. Steffen G. (2024). Design and engineering of bispecific antibodies: insights and practical considerations. Front. Bioeng. Biotechnol. 12:1352014. doi: 10.3389/fbioe.2024.1352014 38333084 PMC10850309

[B2] Anroop B. N. Shery J. (2016). A simple practice guide for dose conversion between animals and human. J. Basic Clin. Pharm. 7, 27–31. doi: 10.4103/0976-0105.177703 27057123 PMC4804402

[B3] Anroop N. Mohamed Aly M. Shery J. (2018). Dose translation between laboratory animals and human in preclinical and clinical phases of drug development. Drug Dev. Res. 79, 373–382. doi: 10.1002/ddr.21461 30343496

[B4] Anshu K. Narendra C. Pradip N. (2016). Immunogenicity of biotherapeutics: causes and association with posttranslational modifications. J. Immunol. Res. 2016:1298473. doi: 10.1155/2016/1298473 27437405 PMC4942633

[B5] Bang L. Xiang-Rong Q. Jia-Chen Q. Guan-du W. Wen-Kang Z. Ze-Zheng J. . (2025). Pair combinations of human monoclonal antibodies fully protected mice against bunyavirus SFTSV lethal challenge. PLoS Pathog. 21:e1012889. doi: 10.1371/journal.ppat.1012889 39888973 PMC11785279

[B6] Bronwyn M. G. Shuangyi B. (2021). Building a better antibody through the Fc: advances and challenges in harnessing antibody Fc effector functions for antiviral protection. Hum. Vaccin Immunother. 17, 4328–4344. doi: 10.1080/21645515.2021.1976580 34613865 PMC8827636

[B7] Chantal T. H. Sivan C. (2024). Reducing immunogenicity by design: approaches to minimize immunogenicity of monoclonal antibodies. BioDrugs 38, 205–226. doi: 10.1007/s40259-023-00641-2 38261155 PMC10912315

[B8] Chen K. Cao G. Xu Y. Yang C. He Z. Shan C. . (2026). Early viral load outperforms cytokines and lymphocytes in predicting SFTS prognosis: a dynamic immune profiling study. J. Clin. Virol. 183:105929. doi: 10.1016/j.jcv.2026.105929 41780268

[B9] Chendan C. Jianhua L. Jiaxuan L. Renjin H. Chenghao C. Jinghan X. . (2026). Research progress on antiviral drugs and vaccines for severe fever with thrombocytopenia syndrome. Front. Immunol. 17:1730089. doi: 10.3389/fimmu.2026.1730089 41676135 PMC12886504

[B10] Chenghao C. Jiaxuan L. Yumo Z. Yuwen Z. Renjin H. Yanjun Z. . (2025). Latest advances and prospects in the pathogenesis, animal models, and vaccine research of severe fever with thrombocytopenia syndrome virus. Front. Immunol. 16:1624290. doi: 10.3389/fimmu.2025.1624290 40642074 PMC12241034

[B11] Chuansong Q. Kaixiao N. Dezhen M. Chao S. Lianfeng L. Wenjun Z. . (2025). Molecular mechanism of potently neutralizing human monoclonal antibodies against severe fever with thrombocytopenia virus infection. J. Virol. 99:e00533. doi: 10.1128/jvi.00533-25 40539781 PMC12282080

[B12] Daniele F. Fabrizio M. (2021). Neutralising antibody escape of SARS-CoV-2 spike protein: Risk assessment for antibody-based Covid-19 therapeutics and vaccines. Rev. Med. Virol. 31:e2231. doi: 10.1002/rmv.2231 33724631 PMC8250244

[B13] Du S. Peng R. Xu W. Qu X. Wang Y. Wang J. . (2023). Cryo-EM structure of severe fever with thrombocytopenia syndrome virus. Nat. Commun. 14:6333. doi: 10.1038/s41467-023-41804-7 37816705 PMC10564799

[B14] Eun Byeol G. Jae Hun L. Jeong Haeng C. Na Hyun K. Jong-Il C. Inchan K. . (2024). Enhanced therapeutic potential of antibody fragment via IEDDA-mediated site-specific albumin conjugation. J. Biol. Eng. 18:23. doi: 10.1186/s13036-024-00418-3 38576037 PMC10996255

[B15] Evi B. S. Jonathan M. O. Tzanko R. Dorothy S. Marjorie A. S. (2023). Uses and challenges of antiviral polyclonal and monoclonal antibody therapies. Pharmaceutics 15:1538. doi: 10.3390/pharmaceutics15051538 37242780 PMC10224075

[B16] Fujifilm Toyama Chemical Co L. (2024). Notification of Additional Indication Approval for Antiviral Drug “Avigan^®^ *Tablets” for Severe Fever with Thrombocytopenia Syndrome (SFTS) Virus Infection*. Tokyo, Japan: FUJIFILM Toyama Chemical Co., Ltd. Available online at: https://www.fujifilm.com/fftc/ja/news/332 (Accessed July 26, 2026).

[B17] Halldorsson S. Behrens A-. J. Harlos K. Huiskonen J. T. Elliott R. M. Crispin M. . (2016). Structure of a phleboviral envelope glycoprotein reveals a consolidated model of membrane fusion. Proc. Natl. Acad. Sci. U. S. A. 113, 7154–7159. doi: 10.1073/pnas.1603827113 27325770 PMC4932967

[B18] Haoliang C. Shijing S. Lin C. Zhiyu F. Qian W. Yiwen X. . (2024). Global epidemiology of severe fever with thrombocytopenia syndrome virus in human and animals: a systematic review and meta-analysis. Lancet Reg. Health West Pac. 48:101133. doi: 10.1016/j.lanwpc.2024.101133 39040038 PMC11261768

[B19] Haoran Z. Yu D. (2025). Nanobodies: from discovery to AI-driven design. Biology 14:547. doi: 10.3390/biology14050547 40427736 PMC12109276

[B20] Huanbo T. Wencheng S. Wenyu Z. Pengju W. Michael S. Peijian Z. . (2019). Recent advances in half-life extension strategies for therapeutic peptides and proteins. Curr. Pharm. Des. 24, 4932–4946. doi: 10.2174/1381612825666190206105232 30727869

[B21] Jia-Chen L. Jing Z. Hao L. Li-Qun F. Wei L. (2022). Epidemiology, clinical characteristics, and treatment of severe fever with thrombocytopenia syndrome. Infect. Med. 1, 40–49. doi: 10.1016/j.imj.2021.10.001 38074982 PMC10699716

[B22] Jinge C. Mengze G. Zhihao Z. Xiaosong L. Qi O. Hui F. . (2025). A hidden guardian: the stability and spectrum of antibody-dependent cell-mediated cytotoxicity in COVID-19 response in chinese adults. Vaccines 13:262. doi: 10.3390/vaccines13030262 40266151 PMC11945335

[B23] Josephine B. Michel V. Alex Paddy A. S. Marie J. Camille F. Shevin T. J. . (2025). Adaptive design for phase II/III platform trial of lassa fever therapeutics. Emerg. Infect. Dis. 31, 9–16. doi: 10.3201/eid3102.240251 39983682 PMC11845141

[B24] Jun L. Yaping H. Yiping X. Shuang L. Lianhua K. Yongxiang Z. . (2014). Concurrent measurement of dynamic changes in viral load, serum enzymes, T cell subsets, and cytokines in patients with severe fever with thrombocytopenia syndrome. PLoS One 9:e91679. doi: 10.1371/journal.pone.0091679 24658451 PMC3962368

[B25] Kaori S. Miyuki K. Akiko S. Hideki H. Hideki T. Tadaki S. . (2024). Characterization of antibodies targeting severe fever with thrombocytopenia syndrome virus glycoprotein Gc. Arch. Virol. 169:40. doi: 10.1007/s00705-024-05968-x 38308735

[B26] Kevin R. M. (2022). Catching the breadth of broadly protective antibodies to SARS-CoV-2. Nat. Immunol. 23, 828–829. doi: 10.1038/s41590-022-01225-y 35654852

[B27] Ki Hyun K. Jinhee K. Meehyun K. June Young C. Hyori K. Seungtaek K. . (2019). An anti-Gn glycoprotein antibody from a convalescent patient potently inhibits the infection of severe fever with thrombocytopenia syndrome virus. PLoS Pathog. 15:e1007375. doi: 10.1371/journal.ppat.1007375 30707748 PMC6380599

[B28] Ko S. Jo M. Jung S. T. (2021). Recent achievements and challenges in prolonging the serum half-lives of therapeutic IgG antibodies through fc engineering. BioDrugs 35, 147–157. doi: 10.1007/s40259-021-00471-0 33608823 PMC7894971

[B29] Lanying D. Yang Y. Xiujuan Z. (2021). Neutralizing antibodies for the prevention and treatment of COVID-19. Cell Mol. Immunol. 18, 2293–2306. doi: 10.1038/s41423-021-00752-2 34497376 PMC8424621

[B30] Lei G. Jinsheng W. Yong Z. Lei Z. Yong L. Wanwan M. . (2019). Socioeconomic burden of severe fever with thrombocytopenia syndrome in endemic areas of Anhui Province, eastern China. Zoonoses Public Health 66, 879–885. doi: 10.1111/zph.12634 31334609

[B31] Leng Y. Mu H-. Z. Cui N. Fang L-. T. Wang R. Ni X-. Y. . (2026). Molecular evolution and spatial transmission of severe fever with thrombocytopenia syndrome virus. Nat. Commun. 17:4499. doi: 10.1038/s41467-026-71008-8 41896574 PMC13187474

[B32] Li Y. Li Z. Lina S. Jing L. Wei W. Chuan L. . (2012). Critical epitopes in the nucleocapsid protein of SFTS virus recognized by a panel of SFTS patients derived human monoclonal antibodies. PLoS One 7:e38291. doi: 10.1371/journal.pone.0038291 22719874 PMC3373585

[B33] Liming L. (2017). Pharmacokinetics of monoclonal antibodies and Fc-fusion proteins. Protein Cell 9, 15–32. doi: 10.1007/s13238-017-0408-4 28421387 PMC5777971

[B34] Marwah K. Chieh-Wen L. Shirit E. (2023). Preparing for the next viral threat with broad-spectrum antivirals. J. Clin. Invest. 133:e170236. doi: 10.1172/JCI170236 37259914 PMC10232003

[B35] Mikhail S. K. Irina V. K. Viktoriya V. D. Kristina O. B. Pavel K. K. Volker S. (2025). Trends and challenges in bispecific antibody production. J. Chromatogr. A. 1744:465722. doi: 10.1016/j.chroma.2025.465722 39884073

[B36] Min W. Weilong T. Jun L. Liqun F. Ming Y. (2022). The endless wars: severe fever with thrombocytopenia syndrome virus, host immune and genetic factors. Front. Cell Infect. Microbiol. 12:808098. doi: 10.3389/fcimb.2022.808098 35782112 PMC9240209

[B37] Mingkai W. Tianlei Y. Yanling W. (2024). Single-domain antibodies as therapeutics for solid tumor treatment. Acta Pharm. Sin. B 14, 2854–2868. doi: 10.1016/j.apsb.2024.03.016 39027249 PMC11252471

[B38] Nathen E. B. Jaclyn A. K. Ashley E. S. Alan D. T. B. David W. C. B Virginia B. . (2020). Baseline mapping of severe fever with thrombocytopenia syndrome virology, epidemiology and vaccine research and development. NPJ Vaccines 5:111. doi: 10.1038/s41541-020-00257-5 33335100 PMC7746727

[B39] Nehad S. E. S. Jordan H. C. Lisanne M. S. Haibo Y. (2024). Nanobody engineering: computational modelling and design for biomedical and therapeutic applications. FEBS Open Bio 15, 236–253. doi: 10.1002/2211-5463.13850 PMC11788755 38898362

[B40] Ole A. M. Sui Ching O. Simon L. Anders D. Imke R.-J. Niels Frederik D.-H. . (2021). Programmable half-life and anti-tumour effects of bispecific T-cell engager-albumin fusions with tuned FcRn affinity. Commun. Biol. 4:310. doi: 10.1038/s42003-021-01790-2 33686177 PMC7940400

[B41] Petra G. Reinhard D. Jochen S. (2014). Trends in upstream and downstream process development for antibody manufacturing. Bioengineering 1, 188–212. doi: 10.3390/bioengineering1040188 28955024

[B42] Praveen R. Sanjana M. Vijay Kumar P. (2025). Exploring immunotherapy to control human infectious diseases. Adv. Protein Chem. Struct. Biol. 144, 389–429. doi: 10.1016/bs.apcsb.2024.10.010 39978973

[B43] Qerqez A. N. Hoffmann K. Lee A. G. Pareek S. Hager K. Mishra A. K. . (2025). Selective decoupling of IgG1 binding to viral Fc receptors restores antibody-mediated NK cell activation against HCMV. Cell Rep. 44:116593. doi: 10.1016/j.celrep.2025.116593 41343276 PMC12831531

[B44] Ramdani Y. Lamamy J. Watier H. Gouilleux-Gruart V. (2022). Monoclonal antibody engineering and design to modulate fcrn activities: a comprehensive review. Int. J. Mol. Sci. 23:9604. doi: 10.3390/ijms23179604 36077002 PMC9455995

[B45] Robin E. W. Angela N. Chloé A. Aurélie M. Jana S. Sofie P. . (2024). Preclinical immunogenicity risk assessment of biotherapeutics using CD4 T cell assays. Front. Immunol. 15:1406040. doi: 10.3389/fimmu.2024.1406040 38863708 PMC11165089

[B46] Sabue M. Lori E. D. Richard D. T. Jr, Olivier, T. M. Michael P. Daniel M. . (2019). A randomized, controlled trial of ebola virus disease therapeutics. N. Engl. J. Med. 381, 2293–2303. doi: 10.1056/NEJMoa1910993 31774950 PMC10680050

[B47] Shannon R.-S. Minakshi N. Nihal A. (2007). Dose translation from animal to human studies revisited. FASEB J. 22, 659–661. doi: 10.1096/fj.07-9574LSF 17942826

[B48] Suemori K. Saijo M. Yamanaka A. Himeji D. Kawamura M. Haku T. . (2021). A multicenter non-randomized, uncontrolled single arm trial for evaluation of the efficacy and the safety of the treatment with favipiravir for patients with severe fever with thrombocytopenia syndrome. PLoS Negl. Trop. Dis. 15:e0009103. doi: 10.1371/journal.pntd.0009103 33617533 PMC7899362

[B49] Su-Jin P. Young-Il K. Mark Anthony C. Eun-Ha K. Se-Mi K. Kwang-Min Y. . (2022). Infection route impacts the pathogenesis of severe fever with thrombocytopenia syndrome virus in ferrets. Viruses 14:1184. doi: 10.3390/v14061184 35746656 PMC9227493

[B50] Vicki S. Esteban C. Barbaros E. Veysel K. (2019). Current advancements in addressing key challenges of therapeutic antibody design, manufacture, and formulation. Antibodies 8:36. doi: 10.3390/antib8020036 31544842 PMC6640721

[B51] Wang L. Wang J. Zhang J. Wang X. Cao W. Yang L. . (2025). Weakened invasion of severe fever with thrombocytopenia syndrome bunyavirus beneficial to its immune escape by multi-site mutation of glycoprotein. Mol. Genet. Genomics 300:109. doi: 10.1007/s00438-025-02319-6 41273444

[B52] Wang Q. Li H. Jian F. Han A. Liu Y. Liu J. . (2026). Human monoclonal antibodies that target the SFTSV glycoprotein Gn head from four neutralizing epitope groups. Cell Rep. 45:117248. doi: 10.1016/j.celrep.2026.117248 41955097

[B53] William R. S. (2017). Current progress in innovative engineered antibodies. Protein Cell 9, 86–120. doi: 10.1007/s13238-017-0457-8 28822103 PMC5777977

[B54] Xiaohan G. Yi W. Ying X. Na X. Guobo S. (2023). Revolutionizing cancer immunotherapy: unleashing the potential of bispecific antibodies for targeted treatment. Front. Immunol. 14:1291836. doi: 10.3389/fimmu.2023.1291836 38106416 PMC10722299

[B55] Xiaoli W. Abulimiti M. Yanfang Z. Zhiying W. Tao Z. Liyan F. . (2024). Identification and characterization of three monoclonal antibodies targeting the SFTSV glycoprotein and displaying a broad spectrum recognition of SFTSV-related viruses. PLoS Negl. Trop. Dis. 18:e0012216. doi: 10.1371/journal.pntd.0012216 38848311 PMC11161016

[B56] Xilin W. Linjing Z. Shengjian L. Zhen C. Yaxin W. Qingcui Z. . (2025). A rationally designed cocktail of nanobodies elicited by heterologous vaccination confers protection against SFTSV in preclinical models. Sci. Transl. Med. 17:eady9025. doi: 10.1126/scitranslmed.ady9025 41259535

[B57] Xilin W. Yanlei L. Bilian H. Xiaohua M. Linjing Z. Nan Z. . (2020). A single-domain antibody inhibits SFTSV and mitigates virus-induced pathogenesis in vivo. JCI Insight 5:e136855. doi: 10.1172/jci.insight.136855 32641581 PMC7406269

[B58] Xiling G. Li Z. Wenshuai Z. Ying C. Xiaoyan Z. Xian L. . (2013). Human antibody neutralizes severe Fever with thrombocytopenia syndrome virus, an emerging hemorrhagic fever virus. Clin. Vaccine Immunol. 20, 1426–1432. doi: 10.1128/CVI.00222-13 23863504 PMC3889583

[B59] Xinhan Y. Chunling J. Shuzhao J. Shuchen C. Youwang L. Lina L. . (2026). Favipiravir's clinical potential for treating severe fever with thrombocytopenia syndrome (SFTS): a narrative review. Virology 617:110799. doi: 10.1016/j.virol.2026.110799 41587499

[B60] Xuanxiu R. Jiawen S. Wenhua K. Feiyang Y. Bingjie W. Yong W. . (2024). A broadly protective antibody targeting glycoprotein Gn inhibits severe fever with thrombocytopenia syndrome virus infection. Nat. Commun. 15:7009. doi: 10.1038/s41467-024-51108-z 39147753 PMC11327358

[B61] Yingda X. Dongdong W. Bruce M. Tony R. Ning L. Dingjiang L. . (2018). Structure, heterogeneity and developability assessment of therapeutic antibodies. MAbs 11, 239–264. doi: 10.1080/19420862.2018.1553476 30543482 PMC6380400

[B62] Yulan S. Cong J. Faxian Z. Xianjun W. Mifang L. Quanfu Z. . (2012). Host cytokine storm is associated with disease severity of severe fever with thrombocytopenia syndrome. J. Infect. Dis. 206, 1085–1094. doi: 10.1093/infdis/jis452 22904342

[B63] Zhen C. Dan G. Liying L. Junqing S. Gen Z. Xue Z. . (2024). Bispecific antibodies targeting two glycoproteins on SFTSV exhibit synergistic neutralization and protection in a mouse model. Proc. Natl. Acad. Sci. U. S. A. 121:e2400163121. doi: 10.1073/pnas.2400163121 38830098 PMC11181109

[B64] Zhen-Dong Y. Jian-Gong H. Qing-Bin L. Chen-Tao G. Ning C. Wei P. . (2016). The prospective evaluation of viral loads in patients with severe fever with thrombocytopenia syndrome. J. Clin. Virol. 78, 123–128. doi: 10.1016/j.jcv.2016.03.017 27062673

[B65] Zuben E. Vibha S. Sathy B.-I. Narendra C. (2023). Understanding preclinical and clinical immunogenicity risks in novel biotherapeutics development. Front. Immunol. 14:1151888. doi: 10.3389/fimmu.2023.1151888 37251396 PMC10213690

